# Expression of lysophosphatidic acid acyltransferase beta (LPAAT-*β*) in ovarian carcinoma: correlation with tumour grading and prognosis

**DOI:** 10.1038/sj.bjc.6602528

**Published:** 2005-04-19

**Authors:** S Niesporek, C Denkert, W Weichert, M Köbel, A Noske, J Sehouli, J W Singer, M Dietel, S Hauptmann

**Affiliations:** 1 Institute of Pathology, Charité University Hospital, Campus Mitte, Schumannstr. 20/21, 10117 Berlin, Germany; 2 Institute of Pathology, Martin-Luther-University Halle-Wittenberg, Magdeburger Str. 14, 06112 Halle (Saale), Germany; 3 Department of Gynecology and Obstetrics, Charité University Hospital, Campus Virchow Klinikum, Augustenburger Platz 1, 13353 Berlin, Germany; 4 Cell Therapeutics Inc., 501 Elliott Avenue West, Suite 400, Seattle, WA 98119, USA

**Keywords:** LPAAT-*β*, ovarian cancer, phosphatidic acid

## Abstract

Lysophosphatidic acid acyltransferase beta (LPAAT-*β*) is an enzyme involved in lipid biosynthesis whose role in tumour progression has been of emerging interest in the last few years. We investigated the expression of LPAAT-*β* by reverse transcriptase–polymerase chain reaction and immunohistochemistry in 10 ovarian cell lines as well as in a cohort of 106 ovarian tumours and normal ovaries. Lysophosphatidic acid acyltransferase beta mRNA was found in all cell lines and ovarian tumours examined. Expression of LPAAT-*β* protein was significantly increased in ovarian carcinomas compared to benign ovarian tissue (*χ*^2^ test *P*-value=0.001, Kruskal–Wallis test *P*-value <0.0001). Furthermore, LPAAT-*β* expression was positively associated with higher tumour grade (*P*=0.044), higher mitotic index (*P*<0.0001) and tumour stage (*P*=0.032). Expression of LPAAT-*β* was significantly linked to reduced overall survival time (*P*=0.024) as well as to shorter progression-free survival time (*P*=0.012) in patients younger than 60 years. Our study shows that LPAAT-*β* is upregulated in ovarian cancer and is more prevalent in poorly differentiated tumours. In addition, LPAAT-*β* expression is a predictor of a worse prognosis in patients younger than 60 years. Further studies are needed to investigate if LPAAT-*β* may serve as a therapeutic target for certain subgroups of patients.

Ovarian carcinoma, although constituting only about 5% of all malignancies of the female genital tract, is still the gynaecological cancer with the highest mortality (Jemal *et al*, 2004). This is mainly due to the usually late detection of this neoplasm. In contrast to breast or cervical cancer, no sufficient screening methods exist for ovarian carcinoma and in contrast to endometrial carcinoma, it is mostly asymptomatic in early stages. Thus, most patients are diagnosed with stage III or IV carcinomas that have already spread to adjacent anatomic structures in the peritoneal cavity. In these stages, chemotherapy in addition to radical surgery is the therapeutic option (Almadrones, 2003; Pfisterer *et al*, 2003). Therefore, research on the pathogenetic basis of this neoplasm might be of great value for the development of new molecular therapies, which specifically target molecules that enhance tumorigenic properties.

Lysophosphatidic acid acyltransferase beta (LPAAT-*β*) is an enzyme that has been of growing interest in tumour research in the last few years. It converts intracellular lysophosphatidic acid (LPA) to phosphatidic acid (PA). Apart from its counterpart LPA that is already well known as a stimulator of cell proliferation, migration and survival and whose role in tumorigenesis, especially in ovarian cancer, has been extensively examined (Mills and Moolenaar, 2003), PA has emerged as another bioactive phospholipid important in cancer biology because of its implication in a variety of signal transduction pathways (English, 1996). The LPAAT family comprises several members with the most important being LPAAT-*α* and LPAAT-*β* (Eberhardt *et al*, 1997; West *et al*, 1997; Aguado and Campbell, 1998; Li *et al*, 2003). While LPAAT-*α* is expressed in all human tissues and is therefore thought to have a housekeeping function, LPAAT-*β* is differentially expressed, with the highest levels in liver and heart but also in steroid hormone-dependent tissues such as prostate, endometrium and ovary (West *et al*, 1997; Leung, 2001).

In this study, we investigated the expression of LPAAT-*β* by reverse transcriptase–polymerase chain reaction (RT–PCR) and immunohistochemistry in 10 ovarian cell lines as well as in a cohort of 106 ovarian tumours and normal ovaries. The aim of our study was to evaluate the prognostic role of LPAAT-*β* expression and its association with clinicopathological factors.

## PATIENTS AND METHODS

### Cell lines

The human ovarian carcinoma cell lines OVCAR-3, SKOV-3, MDAH-2774 and CAOV-3 have been isolated from ovarian adenocarcinomas and were obtained from the American Type Culture Collection (ATCC, Rockville, MD, USA). OAW-42 and A27/80 have been established from adenocarcinomas of the ovary, and were from the European Collection of Cell Cultures (ECACC, Salisbury, UK). EFO-27 has been established from an ovarian adenocarcinoma and was obtained from the German Collection of Microorganisms and Cell Cultures (DSM, Braunschweig, Germany). The cell line ES-2 has been isolated from a poorly differentiated ovarian clear-cell carcinoma and was from ATCC. PA-1 has been established from an ovarian teratocarcinoma and was from ATCC. HOSE, a normal ovarian surface epithelium cell line, which has been immortalised by HPV, was a kind gift from Dr SW Tsao (Universtity of Hong-Kong, Department of Anatomy) (Tsao *et al*, 1995). Cell lines were cultured in Dulbecco's modified Eagle's medium supplemented with 10% fetal bovine serum.

### Polymerase chain reaction

Tissue from ovarian carcinomas was dissected by a senior pathologist in the operating room from surgical specimens sent for frozen section analysis and was immediately frozen in liquid nitrogen and stored at −80°C until analysis. Tissue samples were homogenised and total RNA was extracted with the RNeasy Kit. Residual DNA was digested with DNase. For polymerase chain reaction (PCR) of RNA, cDNA was made by reverse transcription. Polymerase chain reaction was performed following the subsequent protocol: cycling conditions were 35 cycles of denaturation, annealing and extension (94°C for 60 s, 55°C for 60 s, 72°C for 60 s). Primers used were human LPAAT-*β* sense 5′-CGCTCTAGCACTGCCATGAC-3′ and LPAAT-*β* antisense 5′-CCAGCACCTGCAATGTGACT-3′ (generating a 253-bp band) and human GAPDH sense 5′-CCATGGCACCGTCAAGGCTG-3′ and GAPDH antisense 5′-GCCATGTGGGCCATGAGGTC-3′ (generating an 828-bp band).

### Study population

Immunohistochemical analysis was performed retrospectively on tissue samples collected from 106 patients who underwent surgery for diagnostic or therapeutic purpose at the Charité University Hospital Berlin, Germany and the RWTH Aachen, Germany between 1989 and 2000. This study has been approved by the ethics committee of the Charité Hospital. Data on histology, tumour size, nodal status and FIGO stage were extracted from the pathological report at primary diagnosis. The carcinomas were graded according to the Silverberg grading system, which takes account of nuclear polymorphism, mitotic figure count and architectural features (Shimizu *et al*, 1998). For statistical evaluation and survival analysis, only the cases of invasive ovarian carcinoma were included.

Follow-up data on overall survival were available for all patients. Overall survival was defined as the time between diagnosis and death. Data on progression-free survival were available for 51 of 76 (67%) patients. Progression-free survival was defined as the time between diagnosis and the first clinical or pathological evidence of local or distant disease recurrence.

### Immunohistochemistry

Immunohistochemistry was performed using standard procedures. For detection of LPAAT-*β* on tissue samples, we used a mouse monoclonal antibody against human LPAAT-*β*, which was from JW Singer, Cell Therapeutics, Seattle, USA (Bonham *et al*, 2003). For antigen retrieval, slides were boiled for 5 min in 0.01 M sodium citrate buffer, pH 6.0, in a pressure cooker. Slides were incubated with the primary antibody diluted 1 : 200 in antibody diluent solution (Zymed, San Francisco, CA, USA) for 20 min at room temperature and then at 4°C overnight. After washing slides in TBS, a streptavidin-biotin system was applied according to a standard protocol with standard antibody dilutions as provided by the manufacturer (BioGenex, San Ramon, CA, USA). For colour development, a fast red system (Sigma, Deisenhofen, Germany) was used. After colour development was stopped, slides were coverslipped using Aquatex (Merck, Gernsheim, Germany).

### Evaluation of immunohistochemical staining

Immunohistochemical staining was evaluated by two pathologists (CD and SN) who were blinded towards patient's characteristics and outcome. For semiquantitative analysis of staining, an immunoreactivity scoring system (IRS) was applied. For this purpose, the mean number of cells stained (0=no cells stained, 1=less than 10% of cells stained, 2=11–50% of cells stained, 3=51–80% of cells stained, 4=more than 80% of cells stained) as well as staining intensity (0=negative, 1=weak, 2=moderate, 3=strong) was evaluated. Subsequently, the respective score for each case was calculated by multiplication of these two parameters. Those cases, in which disagreement in IRS score evaluation between both observers was evident, were discussed using a multiheaded microscope, until agreement was achieved. For statistical analysis, cases with an IRS of 0–6 were grouped in one group (‘LPAAT-*β*-negative’) and were compared to cases with an IRS of 7–12 (‘LPAAT-*β*-positive’).

### Statistical analysis

Association between clinicopathological parameters and expression of LPAAT-*β* was assessed using either *χ*^2^ test, *χ*^2^ test for trends, Fisher's exact test, Kruskal–Wallis test or Mann–Whitney test. Survival curves were established by the Kaplan–Meier method and compared by applying a log-rank test. Generally, *P*-values smaller than 0.05 were considered significant. For all statistical procedures, SPSS v10.0 software was used.

## RESULTS

### Expression of LPAAT-*β* mRNA in ovarian carcinoma cell lines

**Figure 1:**
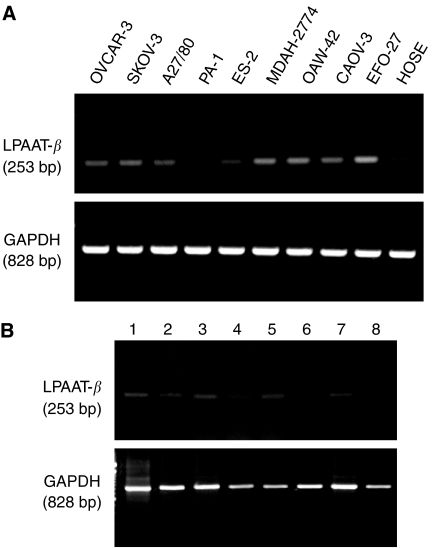
Expression of LPAAT-*β* mRNA in eight ovarian carcinoma cell lines, one ovarian teratocarcinoma cell line and one normal ovarian surface epithelium cell line (**A**). Expression of LPAAT-*β* mRNA in eight cases of ovarian tissue. Histological diagnoses: (1) endometrioid carcinoma, G2; (2) serous carcinoma, G3; (3) benign teratoma; (4) serous carcinoma, G2; (5) normal ovary; (6) serous carcinoma, G3; (7) serous borderline tumour; (8) clear-cell carcinoma (**B**). Expression of LPAAT-*β* and GAPDH was determined by RT–PCR. bp=base pairs.

To investigate expression of LPAAT-*β* mRNA in ovarian cancer, we studied eight ovarian adenocarcinoma cell lines as well as one ovarian teratocarcinoma cell line by RT–PCR. In addition, an immortalised normal ovarian surface epithelium cell line (HOSE) was examined. All cell lines expressed LPAAT-*β* mRNA constitutively. Lysophosphatidic acid acyltransferase beta mRNA was increased in most cancer cell lines compared to the normal ovarian surface epithelium cell line HOSE. Expression level of GAPDH, the positive control, was similar in all cell lines (Figure 1A).

### Expression of LPAAT-*β* mRNA in ovarian carcinomas

We investigated five ovarian carcinomas, one borderline tumour, one benign ovarian teratoma and one normal ovary (Figure 1B). A moderate expression of LPAAT-*β* mRNA was detected in five cases. Three cases, one G2 and one G3 serous carcinoma, and one clear-cell carcinoma, showed only weak expression of LPAAT-*β* mRNA. Expression level of GAPDH, the positive control, was comparable in all cases.

### Clinical and pathological characteristics of patients with ovarian lesions

As our results have shown that LPAAT-*β* mRNA was present in most samples from ovarian carcinomas, we further studied the LPAAT-*β* protein expression by immunohistochemistry in ovarian tissue of 106 patients. The median age at surgery was 57 years (range 28–85 years). In all, 76 (71%) patients had an invasive ovarian carcinoma, 16 (15%) had a borderline tumour, seven (7%) had a benign cystadenoma and seven (7%) had normal ovaries. Of the invasive carcinomas, 39 (51%) were of the serous type, seven (9%) mucinous, 11 (15%) endometrioid, four (5%) clear cell, three (4%) transitional cell and 12 (16%) undifferentiated carcinomas. Carcinomas were well differentiated (G1) in 18 (23%) cases, moderately differentiated (G2) in 31 (41%) cases and poorly differentiated (G3) in 27 (35%) cases. Tumour stage was pT1 in 15 (20%) cases, pT2 in 13 (17%) cases and pT3 in 48 (63%) cases. Data on the nodal status were available for 46 (60%) patients of whom 25 (54%) patients had no lymph node metastasis and 21 (46%) have been classified as pN1. Four (5%) patients in our study group had known distant metastases (pM1) at the time of diagnosis. In all, 16 (21%) patients were staged as FIGO I, 11 (15%) as FIGO II and 45 (59%) as FIGO III. The four (5%) patients with distant metastases were classified as FIGO stage IV.

Overall survival of ovarian carcinoma patients ranged from 0.3 to 122 months with a median survival of 35 months. A total of 35 (46%) patients died during follow-up. A total of 26 of 51 (51%) patients had clinical or pathological evidence of disease recurrence during follow-up. In five (19%) of these 26 patients, disease recurrence was clinically manifest as distant metastasis, while 21 patients (81%) had a local recurrence.

### Expression of LPAAT-*β* in ovarian tissue

**Figure 2:**
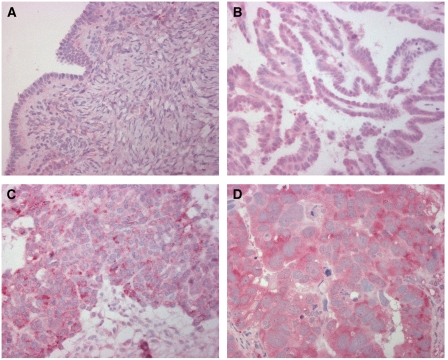
Immunohistochemical detection of LPAAT-*β* expression in a normal ovary as well as in ovarian carcinomas. Normal ovarian surface epithelium with weak expression of LPAAT-*β* (**A**). Serous ovarian carcinoma (G1) with moderate expression of LPAAT-*β* (**B**). Transitional cell ovarian carcinoma (G3) with strong expression of LPAAT-*β* (**C**). Serous ovarian carcinoma (G3) with strong expression of LPAAT-*β* (**D**). In all samples shown, a weak stromal expression of LPAAT-*β* is evident.

**Figure 3:**
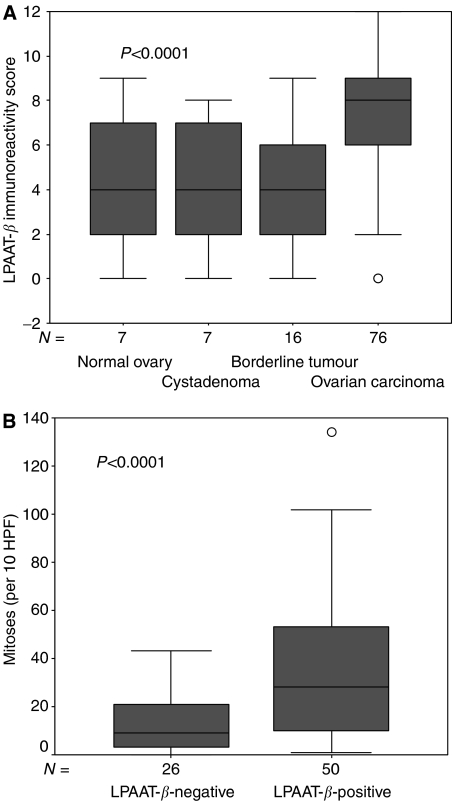
Median immunoreactivity score for LPAAT-*β* staining in normal ovaries, cystadenomas, borderline tumours and ovarian carcinomas (*P*=*P*-value, Kruskal–Wallis test) (**A**). Median number of mitoses (per 10 HPF) in tumours positive and negative for LPAAT-*β* (*P*=*P*-value Mann–Whitney test) (**B**). *N*=number of cases; HPF=high-power field.

Immunoreactivity for LPAAT-*β* was detectable in a subset of ovarian carcinomas as well as in benign ovarian tissue. Lysophosphatidic acid acyltransferase beta was expressed in a cytoplasmatic staining pattern in tumour cells (Figure 2), which is consistent with previous demonstration of LPAAT-*β* localisation to the endoplasmic reticulum (Leung, 2001). We found a positive staining in 50 (66%) ovarian carcinomas, whereas three (19%) borderline tumours, two (29%) benign cystadenomas and two (29%) normal ovaries were LPAAT-*β*-positive (*χ*^2^ test *P*-value=0.001; Table 1). Lysophosphatidic acid acyltransferase beta immunoreactivity score was significantly higher in ovarian carcinomas than in benign ovarian tissue, which comprises borderline tumours, cystadenomas and normal ovarian surface epithelium, as determined with the Kruskal–Wallis test (*P*<0.0001). Median LPAAT-*β* immunoreactivity score was 8 in the cases of ovarian carcinoma, while it was 4 in the cases with borderline tumours, cystadenomas or normal ovarian surface epithelium (Figure 3A).

In addition, in most cases (96%), weak LPAAT-*β* immunoreactivity was found in the stroma of ovarian tumours and normal ovaries where a homogenous staining pattern was observed.

### Association of LPAAT-*β* expression with clinicopathological parameters

Malignant tumours are characterised by clinicopathological parameters such as tumour stage and tumour grade. An association of a certain marker with these parameters could possibly point to a tumourbiological function of the protein.

We found a significant association of LPAAT-*β* expression with tumour grading (*P*=0.044) comparing low-grade cases (G1 and G2) with high-grade cases (G3) (Table 2). As the Silverberg grading system includes nuclear polymorphism, mitotic frequency and architectural features, we determined associations of LPAAT-*β* immunoreactivity with these factors. Expression of LPAAT-*β* showed a significant positive correlation with mitotic figure count (per 10 HPF), as determined by the Mann–Whitney test (*P*<0.0001) (Figure 3B). No association of the expression of the enzyme could be observed with nuclear polymorphism or architectural features.

A significant positive association (*P*=0.032) of LPAAT-*β* expression with tumour stage could be established. Tumours of the pT2 and pT3 stages were more frequently positive for LPAAT-*β* than tumours of the pT1 stage (Table 2). Significant associations of LPAAT-*β* expression with neither nodal status nor state of distant metastasis could be observed. Also, no significant association with FIGO stage was evident.

Consistent with the association of LPAAT-*β* expression and tumour grade, we found that undifferentiated carcinomas had a significantly higher LPAAT-*β* immunoreactivity than carcinomas of a distinct histological type (serous, mucinous, endometrioid, transitional cell or clear cell) (*P*=0.049) (Table 2).

Stromal expression of LPAAT-*β* was associated with neither epithelial expression of the protein nor with any clinicopathological parameter.

### Univariate survival analysis

The known prognostic factors in ovarian carcinoma like FIGO stage, state of metastasis as well as tumour grade and age at diagnosis were prognostic factors for overall survival (Table 3) as well as for progression-free survival (Table 4) in our study group.

**Figure 4:**
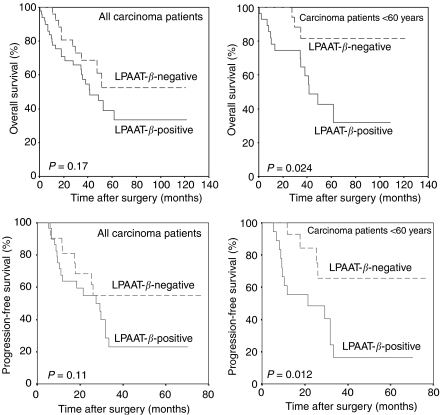
Univariate survival analysis (Kaplan–Meier) for all 76 patients with invasive carcinomas. There was no significant difference in median overall survival in patients with tumours negative or positive for LPAAT-*β* (**A**). For patients younger than 60 years, median overall survival was significantly reduced if they had tumours positive for LPAAT-*β* (**B**). Patients with LPAAT-*β*-positive tumours showed no significant difference in progression-free survival compared to patients with LPAAT-*β*-negative tumours (**C**). Lysophosphatidic acid acyltransferase beta positivity in patients younger than 60 years was a predictor of reduced progression-free survival (**D**). *P*=*P*-value, log-rank test.

Median overall survival was 41.5 months for patients with LPAAT-*β*-positive carcinomas, while it was not reached at the end of follow-up in the group of patients with LPAAT-*β*-negative carcinomas (Figure 4A). Nevertheless, this difference was not statistically significant (*P*=0.17). Similarly, there was no significant difference in progression-free survival (*P*=0.11) between both groups (not shown). However, we found that LPAAT-*β* positivity was a negative prognostic parameter in patients younger than 60 years at the time of diagnosis. As shown in Figure 4B, the median overall survival time for patients younger than 60 years with LPAAT-*β*-positive tumours was 41.5 months, while it was not reached for patients of the same age group with LPAAT-*β*-negative tumours (*P*=0.024). Similarly, progression-free survival was significantly shorter in patients younger than 60 years with LPAAT-*β*-positive tumours (21.3 months *vs* not reached, *P*=0.012; Figure 4D). Patients with LPAAT-*β*-positive tumours had similar frequences of local recurrence and distant metastasis as patients with LPAAT-*β*-negative tumours (not shown). No significant difference in progression-free or overall survival according to LPAAT-*β* was determined for patients older than 60 years (not shown). A preliminary explorative multivariate analysis for the two age groups indicated that the number of cases was too small to obtain reliable results. Survival analysis was not biased by differences in therapy as LPAAT-*β* expression was associated with neither residual tumour mass nor type of chemotherapy (Table 2).

## DISCUSSION

In this study, we systematically determined the expression of LPAAT-*β* mRNA as well as LPAAT-*β* protein *in vivo* and *in vitro* in ovarian carcinomas and in benign ovarian tissue. We found that LPAAT-*β* mRNA was expressed in all ovarian tissue samples and all ovarian cell lines examined. On the protein level, LPAAT-*β* was expressed in most ovarian carcinomas (66%) but only in a minority of benign ovarian samples. Comparative analysis finally revealed a strong increase of LPAAT-*β* expression in invasive ovarian carcinomas compared to normal ovarian surface epithelium, cystadenomas and borderline tumours. Consequently, our study shows an overexpression of LPAAT-*β* in malignant ovarian tissue.

Our finding that LPAAT-*β* expression was upregulated in invasive ovarian carcinomas is consistent with other studies, which reported LPAAT-*β* upregulation in malignant tissue: LPAAT-*β* mRNA expression determined by Northern blot analysis was low to moderate in normal ovarian, breast and prostate tissue while it was high in the corresponding malignant tissue (Leung, 2001). Tissue expression of the protein as determined by immunohistochemistry was elevated in malignant neoplasms of the prostate, breast, lung, colon, cervix, brain as well as ovary compared to normal tissue of the same type (Bonham *et al*, 2003). Furthermore, cell culture experiments showed that prostate carcinoma cell lines expressed LPAAT-*β* at a higher level than normal prostate cell strains and that this correlated with an increase with LPAAT-*β* activity (Bonham *et al*, 2001, 2003).

In our study, we found an increased LPAAT-*β* expression in poorly differentiated tumours as well as an association with tumour size and high mitotic count. This indicates that LPAAT-*β* expression may be involved in the regulation of tumour cell proliferation. This hypothesis, on the functional level, is supported by the results of other authors who reported that transduction and overexpression of the LPAAT-*β* gene in weakly transformed cancer cells *in vitro* lead to the ability to proliferate in low serum (Bonham *et al*, 2001, 2003). These data suggest a cause and effect relationship between LPAAT-*β* function and tumour progression, as opposed to just circumstantial evidence of increased expression in tumours; it appears to be a selective advantage to tumours, possibly by enhanced response to growth factors in cell signalling pathways requiring PA. Phosphatidic acid, the product of LPAAT-*β*, has been shown to play an important role in several signal transduction pathways that stimulate proliferation and prevent apoptosis. Thus, it is necessary for translocation of raf to membranes, a step being essential for raf activation (Andresen *et al*, 2002). Furthermore, PA is involved in mTOR activation (Fang *et al*, 2001), which in turn plays a role in the Akt/PI3K pathway in preventing apoptosis. There is evidence that PA can directly activate ERK (Andresen *et al*, 2002). In addition, overexpression of LPAAT-*β*, but not the alpha isoform, cDNA in *Xenopus* oocytes enhanced both raf- and ras-mediated activation of ERK and associated germinal vessel breakdown (Coon *et al*, 2003). Phosphatidic acid is also implicated in the activation of Rho family proteins like Rac (Chuang *et al*, 1993), RhoA (Lee *et al*, 1998) and Arf (Manifava *et al*, 2001). These small GTPases in regulating the actin cytoskeleton play a role in cellular migration and invasion and, in addition, promote cell cycle progression by regulation of cell cycle proteins like p21 and cyclin D1 (Etienne-Manneville and Hall, 2002). Further messenger functions of PA include EGFR internalisation (Shen *et al*, 2001), vesicle formation (English, 1996; English *et al*, 1996) and calcium homeostasis (English, 1996; English *et al*, 1996). Several of these pathways are activated in ovarian carcinoma as was shown for EGFR, PI3K or Akt (Berchuck *et al*, 1990; Wenham *et al*, 2002; Zhang *et al*, 2003). Enhanced PA signalling in LPAAT-*β*-overexpressing tumours may contribute to this deregulation and result in tumour growth and dedifferentiation. Phosphatidic acid-mediated activation of Rho proteins, which have been shown to be implicated in the progression in ovarian cancer (Horiuchi *et al*, 2003), could enhance invasion, metastasis and proliferation. The hypothesis that PA itself has an impact on ovarian carcinoma tumorigensis is supported by the fact that PA directly stimulates proliferation in ovarian cancer cell lines (Xu *et al*, 1995). Our finding of an increased LPAAT-*β* expression in worse differentiated and more advanced tumours may reflect these biological mechanisms.

Further evidence for an important role of LPAAT-*β* in human cell signalling pathways came from experiments with specific LPAAT-*β* inhibitors. Treatment of smooth muscle cells, in which cell signalling pathways have been well characterised, decreased phosphorylation of ERK, Akt and mTOR (Coon *et al*, 2003). Moreover, LPAAT-*β* inhibition of a variety of human tumour cell lines leads to a proliferation arrest in G_2_–M and subsequent apoptosis (Bonham *et al*, 2003; Coon *et al*, 2003; Hideshima *et al*, 2003). Interestingly, a proapoptotic effect of the LPAAT-*β* inhibitors was found in most tumour cell lines, which also comprised ovarian cancer cell lines, but not in some normal cell types, even when high concentrations of inhibitors were used (Bonham *et al*, 2003; Coon *et al*, 2003). Lysophosphatidic acid acyltransferase beta, which normally is expressed in low levels in most normal tissues, may not be necessary for survival of these normal cells, but the malignant cells may be more dependent on its activity. Thus, a future scenario in cancer therapy might be to use LPAAT-*β* inhibitors for patients with LPAAT-*β*-overexpressing tumours to specifically suppress multiple pathways of neoplastic cell growth.

In the present study, LPAAT-*β* positivity was a predictor of unfavourable outcome with respect to overall and progression-free survival in patients, younger than 60 years. This might indicate that in younger patients, hormonal influences act together with LPAAT-*β* expression to worsen the prognosis. In this context, studies on the impact of oestrogen and progesterone on LPAAT-*β* regulation would be of great interest. Lysophosphatidic acid acyltransferase beta expression might be of prognostic value only for certain subgroups of patients, as the expression of the enzyme was no prognostic marker for overall or progression-free survival in the whole study population. A limitation of our study was the relatively small patient number (*n*=76), which lowers the power of a survival analysis, especially when performed for subgroups. For this reason, we could not perform a multivariate survival analysis for the different age groups.

Lysophosphatidic acid acyltransferase beta is an interesting new target for chemotherapeutical intervention in patients with ovarian carcinoma. Our study shows that LPAAT-*β* is upregulated in ovarian cancer and is more prevalent in less-differentiated tumours. In addition, LPAAT-*β* expression was a predictor of a worse prognosis in patients younger than 60 years. Large-scale prospective and retrospective studies are needed to establish whether LPAAT-*β* expression could be of diagnostic value as a prognostic parameter for certain subgroups of patients. Furthermore, the mechanism of LPAAT-*β* upregulation in tumours remains to be elucidated.

## Figures and Tables

**Table 1:** Expression of LPAAT-*β* in normal ovaries and different types of ovarian tumours

**Tissue type**	**All cases**	**LPAAT-*β*-negative**	**LPAAT-*β*-positive**	***P*-value**
Invasive carcinoma	76 (100%)	26 (34%)	50 (66%)	
Borderline tumour	16 (100%)	13 (81%)	3 (19%)	
Cystadenoma	7 (100%)	5 (71%)	2 (29%)	
Normal ovary	7 (100%)	5 (71%)	2 (29%)	0.001^a^

a Pearson's *χ*^2^, two-sided.

**Table 2:** Association of LPAAT-*β* expression and various clinicopathological factors

**Characteristic**	**All cases**	**LPAAT-*β*-negative**	**LPAAT-*β*-positive**	***P*-value**
*Histological type*	64 (100%)	25 (39%)	39 (61%)	
Differentiated				
Undifferentiated	12 (100%)	1 (8%)	11 (92%)	0.049^a^

*Histological grade (Silverberg)*				
G1–2	49 (100%)	21 (43%)	28 (57%)	
G3	27 (100%)	5 (19%)	22 (81%)	0.044^a^

*PT*				
pT1	15 (100%)	9 (60%)	6 (40%)	
pT2–3	61 (100%)	17 (28%)	44 (72%)	0.032^a^

*PN*				
pN0	25 (100%)	9 (36%)	16 (64%)	
pN1	21 (100%)	7 (33%)	14 (67%)	1.0^a^

*PM*				
PMX	72 (100%)	24 (33%)	48 (67%)	
pM1	4 (100%)	2 (50%)	2 (50%)	0.6^a^

*FIGO stage*				
I	16 (100%)	8 (50%)	8 (50%)	
II	11 (100%)	2 (18%)	9 (82%)	
III	45 (100%)	14 (31%)	31 (69%)	
IV	4 (100%)	2 (50%)	2 (50%)	0.47^b^

*Residual tumour*				
< 2 cm	26 (100%)	9 (35%)	17 (65%)	
>2 cm	6 (100%)	1 (17%)	5 (83%)	0.64^a^

*Chemotherapy*				
Platinum-based CTX	44 (100%)	16 (36%)	28 (64%)	
Other CTX	4 (100%)	1 (25%)	3 (75%)	
No CTX	7 (100%)	4 (57%)	3 (43%)	0.4^c^

*Age at surgery (years)*				
<60	45 (100%)	17 (38%)	28 (62%)	
>60	31 (100%)	9 (29%)	22 (71%)	0.47^a^

a Fisher's exact test, two-sided.

b *χ*^2^ for trends.

c Pearson's *χ*^2^, two-sided.

**Table 3:** Univariate Kaplan–Meier survival analysis: median overall survival time of all patients with invasive ovarian carcinoma in dependence of clinicopathological factors and LPAAT-*β* expression

**Characteristic**	**No. of cases**	**Median survival time (months)**	**Standard error**	**Log-rank *P*-value**
*LPAAT-β expression*				
Negative	26	Not reached	—	
Positive	50	41.5	8.4	0.17

*Histological type*				
Differentiated	64	51.4	6.6	
Undifferentiated	12	17.8	—	0.24

*PT*				
pT1	15	Not reached	—	
pT2	13	61.6	21.8	
pT3	48	41.2	4.6	0.12

*PN*				
pN0	25	61.6	—	
pN1	21	Not reached	—	0.4

*FIGO stage*				
I	16	Not reached	—	
II	11	52.5	18.4	
III	45	41.5	7.0	
IV	4	1.7	5.3	<0.00001

*Histological grade (Silverberg)*				
G1	18	Not reached	—	
G2	31	47.5	5.4	
G3	27	34.8	5.6	0.013

*Age at surgery (years)*				
<60	45	61.6	—	
>60	31	35.5	15.9	0.044

**Table 4:** Univariate Kaplan–Meier survival analysis: median overall and progression-free survival time of patients with invasive ovarian carcinoma in different age groups in dependence of LPAAT-*β* expression

**Characteristic**	**No. of cases**	**Median survival time (months)**	**Standard error**	**Log-rank *P*-value**
*Overall survival*				
Age <60				
LPAAT-*β* expression				
Negative	17	Not reached	—	
Positve	28	41.5	6.9	0.024

Age >60				
LPAAT-*β* expression				
Negative	9	17.9	1.6	
Positive	22	35.5	15.2	0.29

*Progression-free survival*				
Age < 60				
LPAAT-*β* expression				
Negative	14	Not reached	—	
Positve	18	21.3	14.2	0.012

Age >60				
LPAAT-*β* expression				
Negative	7	17.6	4.8	
Positive	12	29.8	7.5	0.28
